# cIAP-1 Controls Innate Immunity to *C. pneumoniae* Pulmonary Infection

**DOI:** 10.1371/journal.pone.0006519

**Published:** 2009-08-06

**Authors:** Hridayesh Prakash, Daniel Becker, Linda Böhme, Lori Albert, Martin Witzenrath, Simone Rosseau, Thomas F. Meyer, Thomas Rudel

**Affiliations:** 1 Department of Molecular Biology, Max Planck Institute for Infection Biology, Charitéplatz 1, Berlin, Germany; 2 Lehrstuhl für Mikrobiologie Biozentrum, Am Hubland, Würzburg, Germany; 3 Department of Internal Medicine/Infectious Diseases, Charité, Humboldt University, Berlin, Germany; 4 The Campbell Family Institute for Breast Cancer Research, Princess Margaret Hospital, Toronto, Ontario, Canada; Duke University Medical Center, United States of America

## Abstract

The resistance of epithelial cells infected with *Chlamydophila pneumoniae* for apoptosis has been attributed to the induced expression and increased stability of anti-apoptotic proteins called inhibitor of apoptosis proteins (IAPs). The significance of cellular inhibitor of apoptosis protein-1 (cIAP-1) in *C. pneumoniae* pulmonary infection and innate immune response was investigated in cIAP-1 knockout (KO) mice using a novel non-invasive intra-tracheal infection method. In contrast to wildtype, cIAP-1 knockout mice failed to clear the infection from their lungs. Wildtype mice responded to infection with a strong inflammatory response in the lung. In contrast, the recruitment of macrophages was reduced in cIAP-1 KO mice compared to wildtype mice. The concentration of Interferon gamma (IFN-γ) was increased whereas that of Tumor Necrosis Factor (TNF-α) was reduced in the lungs of infected cIAP-1 KO mice compared to infected wildtype mice. *Ex vivo* experiments on mouse peritoneal macrophages and splenocytes revealed that cIAP-1 is required for innate immune responses of these cells. Our findings thus suggest a new immunoregulatory role of cIAP-1 in the course of bacterial infection.

## Introduction

*C. pneumoniae* is a Gram-negative obligate intracellular bacterium responsible for pulmonary infectious diseases. The presence of this pathogen in atheromatous plaques implicates its association with cardiovascular diseases such as atherosclerosis which leads to coronary heart disease, one of the major factors responsible for worldwide mortalities.

*C. pneumoniae* infection starts with the attachment of the virulent and metabolically inert form of the bacteria known as elementary bodies (EB) to epithelial cells. This is followed by a unique bi-phasic life cycle in which EBs differentiate into the non-virulent and metabolic active form called reticulate bodies (RBs) [1]. *C. pneumoniae* infects cell types other than epithelial cells in the course of infection, such as endothelial cells, smooth muscle cells, alveolar and blood macrophages [2]–[5]. Among these, macrophages have gained significant attention in recent years because they engulf these bacteria and transmit them from lungs to peripheral lymphoid tissues for elimination [6]. Macrophages are the key players in the innate immune defense against various intracellular bacterial infections [7]. They reside in almost all tissues constituting the ‘mononuclear phagocyte system’. This complex network enables the immune system to effectively sense the microbial invaders and eliminate them from the body. Upon recognition of pathogen-associated molecular patterns [8] or molecules released by damaged host cells, referred to as “danger signals” [9], macrophages manifest a strong inflammatory response characterized by secretion of various mediators like TNF-α, IFN-γ, IL-8 and nitric oxide. Together these effector molecules confer immune defense against various intracellular bacterial infections including *C. pneumoniae* [10].

During the early phase of infection, *Chlamydia* induce anti-apoptotic pathways conferring resistance of the infected host cells to apoptotic stimuli like TNF-α, cycloheximide, staurosporine, FasL, UV and gamma irradiation [11]–[13]. Recently, it has been shown that resistance of infected cells for apoptosis induction is due to the induced expression and increased stability of anti-apoptotic IAPs, like X-chromosome linked (XIAP), and cellular (cIAPs) inhibitors of apoptosis [14], [15]. IAP proteins, originally found in the baculovirus, are evolutionary conserved from insects to humans and play a principle role in regulating apoptosis [16]. Although several members of the human IAP family of proteins, including XIAP, cIAP-1 and cIAP-2, interact with caspase-3, 7 and 9 and block apoptosis if over expressed in cells [17], [18], the function of IAPs *in vivo* is still unknown. XIAP is probably the only potent direct inhibitor of caspase-3, 7 or 9 [19], but an apoptosis related phenotype has not yet been identified in XIAP knockout mice [20]. A recent report suggests that IAPs are multifunctional signaling devices that influence innate immunity in *Drosophila* [21]. Human cIAP-1 and cIAP-2 may play a role in controlling the tumor necrosis factor pathway since they have been shown to interact with TNF receptor complex components, TRAF1 and TRAF2 [22], [23]. The significance of both, cIAP-1 and cIAP-2 for NF-κB activation has been demonstrated in knockout mice [24]. cIAP-2 knockout mice also displayed an attenuated inflammatory response and profound resistance to LPS in experimental endotoxic shock. Furthermore, cIAP-2 knockout macrophages were reported to be highly susceptible to apoptosis in an LPS-induced proinflammatory environment, indicating that cIAP-2 is a critical factor in maintaining normal innate immune responses [24]. Recently, it has been suggested that XIAP is involved in innate immune responses to control *Listeria* infection in mice [25].

Here we present data demonstrating a compromised immunocompetence of cIAP-1 KO mice against *C. pneumoniae* infection. Macrophages from cIAP-1 KO mice were severely affected in bactericidal innate immune signaling pathways. We propose a new function for inhibitor of apoptosis proteins in the control of infection with obligate intracellular *C. pneumoniae*.

## Results

### Increased sensitivity of cIAP-1 KO mice for *C. pneumoniae* infection

**Figure 1:**
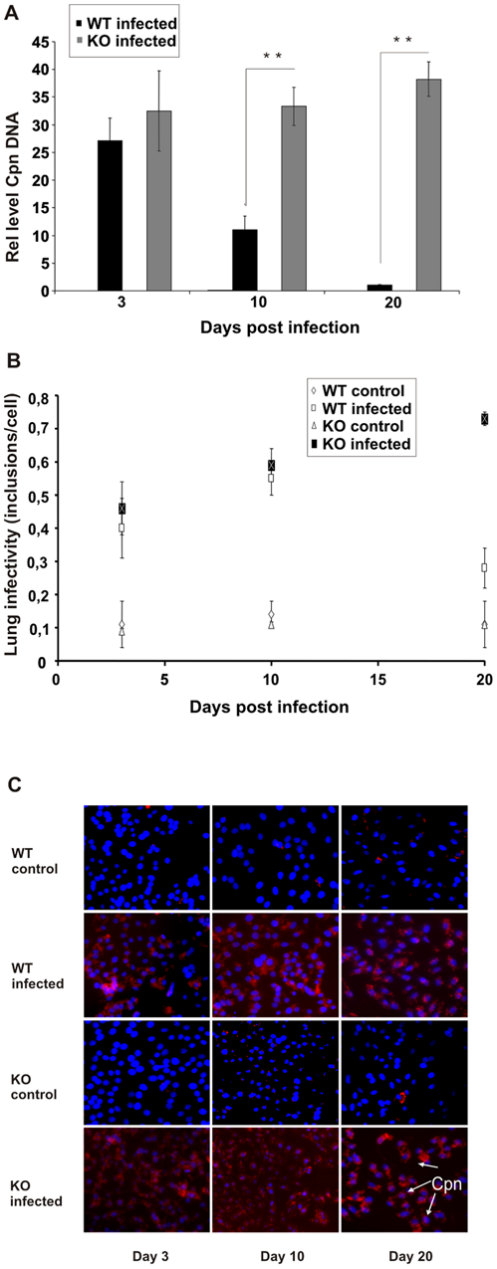
Sensitization of cIAP-1 KO mice for *C. pneumoniae* lung infection. A. Five mice per group were infected with *C. pneumoniae* and the chlamydial *omp*A gene was amplified from the lung tissue of each mouse (as described in Supporting Materials and Methods S1). The relative amount of the *omp*A gene in each lung sample was calculated using GAPDH as an internal control. ** p≤0.01. B. Impaired resolution of *C. pneumoniae* infection from the lungs of cIAP-1 KO mice. Five mice per group were infected with *C. pneumoniae* and infectious bacteria were determined by lung infectivity assays as described in Materials and Methods. HEp2 epithelial cells were infected with lung homogenate at different time points post infection and infectivity was analyzed by quantitative immuofluoresence microscopy. Infectivity was calculated as the number of chlamydial inclusions formed per infected HEp2 cell (cIAP-1 KO infected 0.73±0.023; WT infected 0.28±0.06 at day 20). The data represent the mean±SEM of inclusion numbers per cell from three independent experiments. C. Microscopic images of the chlamydial inclusion formed in HEp2 cell after infection with lung homogenate. The blue color represents the nuclei (Hoechst stain) of the HEp2 cell infected and the red color shows the *Chlamydia* inclusion (Cpn; arrowheads) formed per cell. The red background staining in the control samples is probably due to unspecific antibody aggregate formation.

To investigate if cIAP-1 is involved in the control of chlamydial infection, both wildtype and cIAP-1 KO mice were infected using a non-invasive intratracheal infection method (for details see Materials and Methods). The infection of different mouse organs was monitored by demonstrating the presence of bacterial DNA by nested PCR (Table S1 and S2). Amongst all organs tested, lungs were consistently infected with *C. pneumoniae* at day 3, 10 and 20 post infection (d. p.i) in wildtype and cIAP-1 KO mice (Table S2). Chlamydial inclusions could be detected in infected mouse lungs as early as 3 d.p.i. (Fig. S1). However, quantitative real-time PCR revealed a gradual reduction of bacterial load in lungs of infected wildtype mice, whereas in cIAP-1 KO mice levels remained high (Fig. 1A). In contrast to wildtype mice, cIAP-1 KO mice failed to control the lung infection with *C. pneumoniae.*

To compare the load of viable bacteria in the lung of infected mice, lung infectivity assays were performed. Lungs of non-infected and infected animals were homogenized and infectious chlamydial particles were determined by infecting fresh HEp2 cells and by counting inclusions by automated microscopy. These assays revealed an increase in infectious bacteria in lungs of wildtype infected mice from day 3 to day 10 post-infection which decreased until day 20 post infection (Fig. 1B,C). In contrast to wildtype mice, cIAP-1 KO mice were unable to resolve the infection. During the whole experimental period the amount of infectious bacteria gradually increased (Fig. 1B,C). Taken together, these results demonstrated an increased sensitivity of cIAP-1 KO mice to *C. pneumoniae* lung infection.

### Deregulated inflammatory response in cIAP-1 KO mice

**Figure 2:**
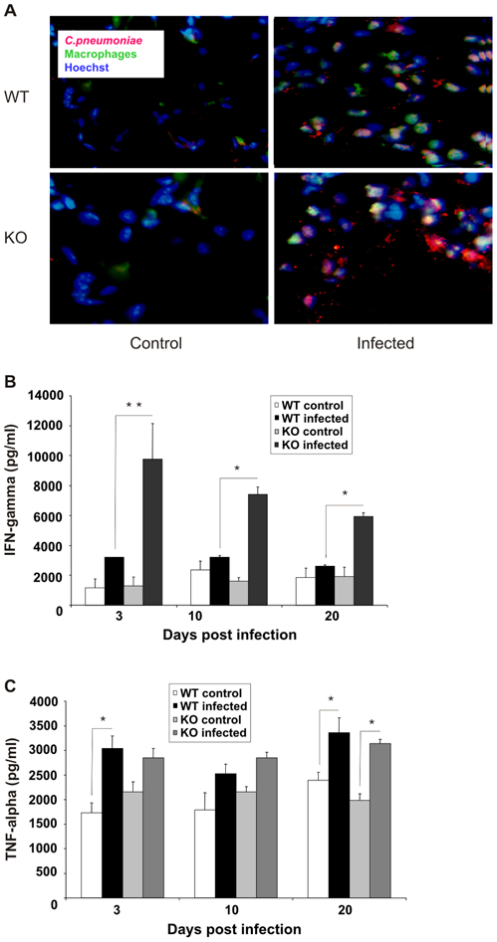
Dysregulated inflammatory responses of cIAP-1 KO mice. A. Reduced numbers of macrophages in lungs of infected cIAP-1 KO mice. The lungs from mice either non-infected or infected for 20 days were excised aseptically and 5 mm pieces were fixed and sectioned. The sections were stained with rabbit anti-*Chlamydia* LPS antibody and Cy3-conjugated secondary antibody, and with anti-CD68 antibody detected with Alexa-488 conjugated secondary antibody to localize *C. pneumoniae* and alveolar macrophages, respectively. The lung epithelial cells were stained with Hoechst and examined microscopically by using a fluorescent microscope under 40×of objective. The figure shows a typical example of at least five independent infections. B, C. Levels of IFN-γ (B) and TNF-α (C) were determined in lung homogenates from wildtype and cIAP-1 KO mice by ELISA at different time points post infection. Data represent the mean±SEM from five independent infection experiments. * p≤0.05; ** p≤0.01.

To obtain first indications of the mechanism underlying the different outcomes of lung infection in wildtype and cIAP-1 KO mice, lungs of infected and non-infected animals were analyzed for production of inflammatory cytokines. Histopathological analyses indicated an increased accumulation and recruitment of inflammatory (CD68+) alveolar macrophages in lungs of wildtype mice in response to *C. pneumoniae* infection (Fig. 2A). These macrophages were located close to bacterial inclusions in infected lungs of wildtype mice. Accumulation of the inflammatory macrophages in lungs of wildtype mice in response to infection indicated a chemotactic response, which was found to be compromised in the infected lung of cIAP-1 KO mice.

We next quantified the inflammatory response by determining IFN-γ and TNF-α titers in lungs of wildtype and cIAP-1 KO mice in response to infection. *C. pneumoniae* infection caused an increase in pulmonary IFN-γ and TNF-α in both wildtype and cIAP-1 KO mice as compared to the non-infected control (Fig. 2B,C,D). Infected cIAP-1 KO mice produced higher pulmonary IFN-γ titers than infected wildtype mice. These data demonstrated a partial de-regulation of the inflammatory response of cIAP-1 KO mice.

### cIAP-1 is required for the production of TNF-α in macrophages

**Figure 3:**
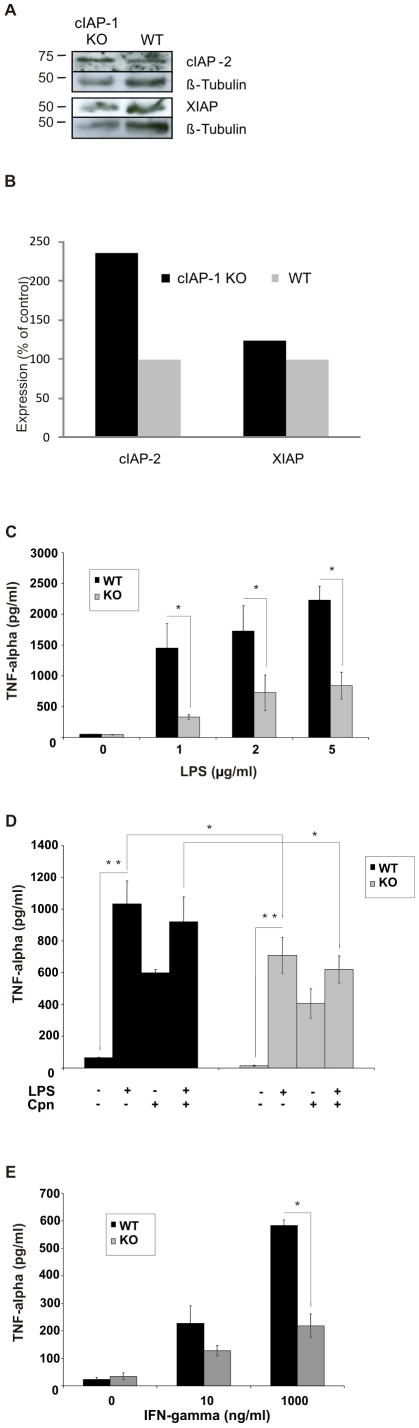
cIAP-1 is required for endotoxin- (LPS) and IFN-γ-induced inflammatory response. A. cIAP-2 is upregulated in the lungs of knockout mice. Lung homogenates from wildtype ad knockout mice were subjected to immunoblot analysis for the quantification of cIAP-2 and XIAP as described in Materials and Methods. β-tubulin was detected as a loading control. B. Densitometric quantification of the data shown in figure 3A normalized for β-tubulin as loading control. C. The isolated peritoneal macrophages from both wildtype and cIAP-1 KO mice were treated with different doses of LPS, and TNF-α was determined in the culture supernatants 48 h post treatment. Data represent the mean±SEM from three independent experiments. D. Impaired TNF-α production in infected cIAP-1 KO macrophages. Macrophages were cultured and infected with *C. pneumoniae* at MOI 1 and/or treated with LPS at 5 µg/ml. TNF-α titers were determined in the culture supernatants 48 h post infection and treatment. Data are presented as the mean±SE from three independent experiments. E. cIAP-1 is required for IFN-γ-mediated production of TNF-α. Peritoneal macrophages from wildtype and cIAP-1 KO mice were isolated and stimulated with different doses of mouse IFN-γ *ex vivo*. The supernatant were collected at 48 h after stimulation and TNF-α titers were quantified by ELISA. Data represent the mean±SEM from two independent experiments. * p≤0.05; ** p≤0.01.

To understand the reason for the dysregulated inflammatory response in cIAP-1 KO mice, innate immune functions of wildtype and cIAP-1 KO macrophages were analyzed. Because increased expression of closely related cIAP-2 might counterbalance the loss of functional cIAP-1 [33], the expression level of cIAP-2 was checked by immuoblot analysis. No increase of cIAP-2 level was observed in macrophages whereas in lung tissue elevated levels of cIAP-2 were observed (Fig. S2, 3A,B). Peritoneal macrophages from both wildtype and cIAP-1 KO mice were stimulated with bacterial LPS (Fig. 3C) and/or infected with *C. pneumoniae* (Fig. 3D). Treatment of wildtype macrophages with increasing doses of LPS (1–5 µg/ml) resulted in a dose-dependent stimulation of TNF-α secretion. cIAP-1 KO macrophages responded similarly to LPS treatment, however, TNF-α titers remained significantly lower than in wildtype macrophages (Fig. 3C). This tendency was also observed if macrophages were infected or stimulated with LPS and infected with *C. pneumoniae* (Fig. 3D). These findings together indicated a possible role of cIAP-1 in the LPS- and infection-induced production of TNF-α in macrophages.

The defects observed in LPS- and infection-induced TNF-α production in cIAP-1 KO macrophages could be restricted to LPS-induced pathways. We therefore stimulated macrophages with IFN-γ, which stimulates TNF-α production via NF-κB independent of LPS [26]. However, cIAP-1 KO macrophages produced significantly less TNF-α compared to wildtype macrophages (Fig. 3E), ruling out a LPS-specific defect in these macrophages.

### cIAP-1 is required for the production of NO in macrophages

**Figure 4:**
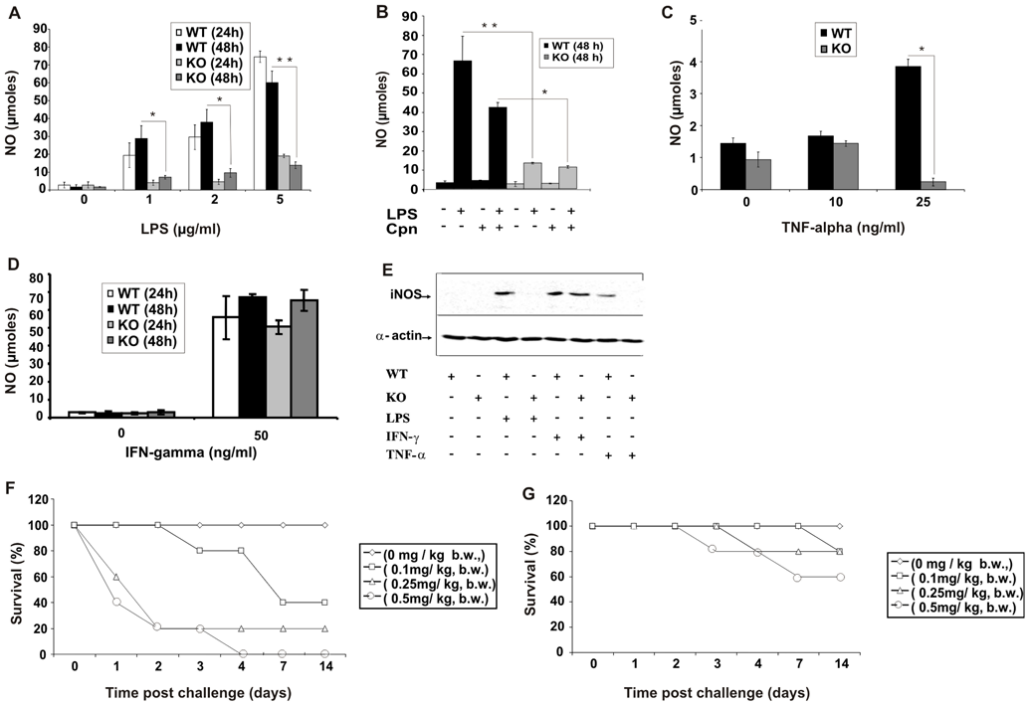
cIAP-1 is required for nitric oxide production in response to LPS and TNF-α in peritoneal macrophages. A. Macrophages from wildtype and cIAP-1 KO mice were stimulated with different doses of LPS and the NO titer was determined in the culture supernatant at the indicated time points post treatment. B. LPS- and infection- (Cpn) induced NO production in cIAP-1 KO and wildtype macrophages. 1×10^4^ peritoneal macrophages were infected with *C. pneumoniae* at an MOI of 1. Cells were treated with LPS 30 min after infection and further incubated at 37°C for 48 h. NO generation was quantified in culture supernatants. C. TNF-α-induced production of NO in cIAP-1 KO and wildtype peritoneal macrophages 24 h post treatment. Both wildtype and cIAP-1 KO macrophages were cultured and stimulated with the indicated doses of TNF-α and NO was measured in the supernatant 24 h post induction. D. IFN-γ-mediated NO production in KO and wildtype macrophages. 1×10^6^ macrophages were stimulated with murine IFN-γ and NO were determined in culture supernatants 48 h later. The data are presented as the mean±SEM from three independent experiments. E. cIAP-1 is required for the induction of iNOS by LPS and TNF-α in mouse macrophages. The cells were incubated with the indicated reagents and the levels of iNOS were determined by western blot of whole cell lysates. α-actin was detected as loading control. F and G. Resistance of cIAP-1 KO mice against LPS-induced endotoxin shock. Mice were treated with different doses of LPS intraperitoneally (from 0.1 to 0.5 mg/kg body weight [b.w.]) and the survival of wildtype (F) and cIAP-1 KO mice (G) was monitored over a 14 day period. Shown are the representative results of one out of three independent experiments. * p≤0.05; ** p≤0.01.

Another important mediator involved in the bactericidal effect of macrophages is nitric oxide (NO) free radical. Treatment of wildtype macrophages by bacterial endotoxin induced the generation of significant amounts of NO in a dose- and time-dependent fashion (Fig. 4A). In contrast, cIAP-1 KO macrophages had a strongly diminished capacity to produce NO upon LPS stimulation. *C. pneumoniae* infection of wildtype macrophages induced only low amounts of NO 24 h post infection (not shown), which was modestly but significantly higher at 48 h post infection in comparison to the uninfected control (Fig. 4B). In contrast, cIAP-1 KO macrophages did not show any increase in NO upon infection during the experimental period (Fig. 4B).

To test whether cIAP-1 is involved in innate stimulation of NO production, we stimulated these macrophages with different doses of TNF-α and IFN-γ, and NO titers were quantified. TNF-treatment of wildtype macrophages induced production of NO in a dose-dependent manner (Fig. 4C). cIAP-1 KO macrophages failed to produce significant levels of NO in response to TNF-α. The lack of NO production by KO macrophages upon TNF-α stimulation was not due to reduced viability of the treated cells (Fig. S3). In contrast to TNF-α, treatment of macrophages with IFN-γ elicited similar NO responses in wildtype and KO macrophages, demonstrating that cIAP-1 KO macrophages have a stimulus-dependent defect in NO signaling (Fig. 4D).

NO is mainly produced by the inducible NO synthase (iNOS) in macrophages [27]. The clear defect of cIAP-1 KO macrophages in NO production prompted us to investigate whether these cells are affected in iNOS expression. Interestingly, LPS or TNF-α treatment induced iNOS expression in wildtype, but not in cIAP KO macrophages (Fig. 4E). In contrast, IFN-γ caused the up-regulation of iNOS in both wildtype and KO (Fig. 4E) ruling out a general defect in iNOS expression in the KO mice. Because cIAP-1 has been implicated in the control of apoptosis, we investigated the effect of these treatments on the induction of apoptosis in wildtype and mutant macrophages. Since none of the treatments elicited a significant increase in the apoptotic population or affected the viability of treated macrophages (Fig. S3), the reduced production of NO by mutant macrophages in response to certain stimuli was not due to increased cell death in these cases. These data suggested that the defect of cIAP KO macrophages in inducing NO originated from a defect in the stimulus dependent up-regulation of iNOS.

### Resistance of cIAP-1 KO mice to endotoxin shock

TNF-α and NO are the key mediators of endotoxin-mediated toxicities. Since cIAP-1 KO macrophages were clearly affected in their production of TNF-α and NO, we expected an increased resistance of these mice to endotoxin-induced toxicities. We therefore determined the dose of lethal endotoxin-induced shock in wildtype and cIAP-1 KO mice. Wildtype mice died in a dose-dependent fashion; for example, a dose of 0.5 mg/kg body weight of bacterial LPS evoked 100% mortality within 96 h post induction of shock in wildtype mice (Fig. 4F). In contrast, 60% of the cIAP-1 KO mice survived treatment with the same dose of LPS (Fig. 4G), demonstrating a significantly increased resistance of these mice for LPS-induced shock. In contrast to the LPS-induced shock, we found no difference in survival and bacterial load in the spleen after i.v. challenge of wildtype and cIAP-1 KO mice with *Salmonella* Typhimurium SL1344 (Fig. S4), suggesting that additional mechanisms besides endotoxin exposure cause toxicity in the case of *Salmonella* infection.

### *C. pneumoniae* infection elicits cytotoxicity on macrophages

**Figure 5:**
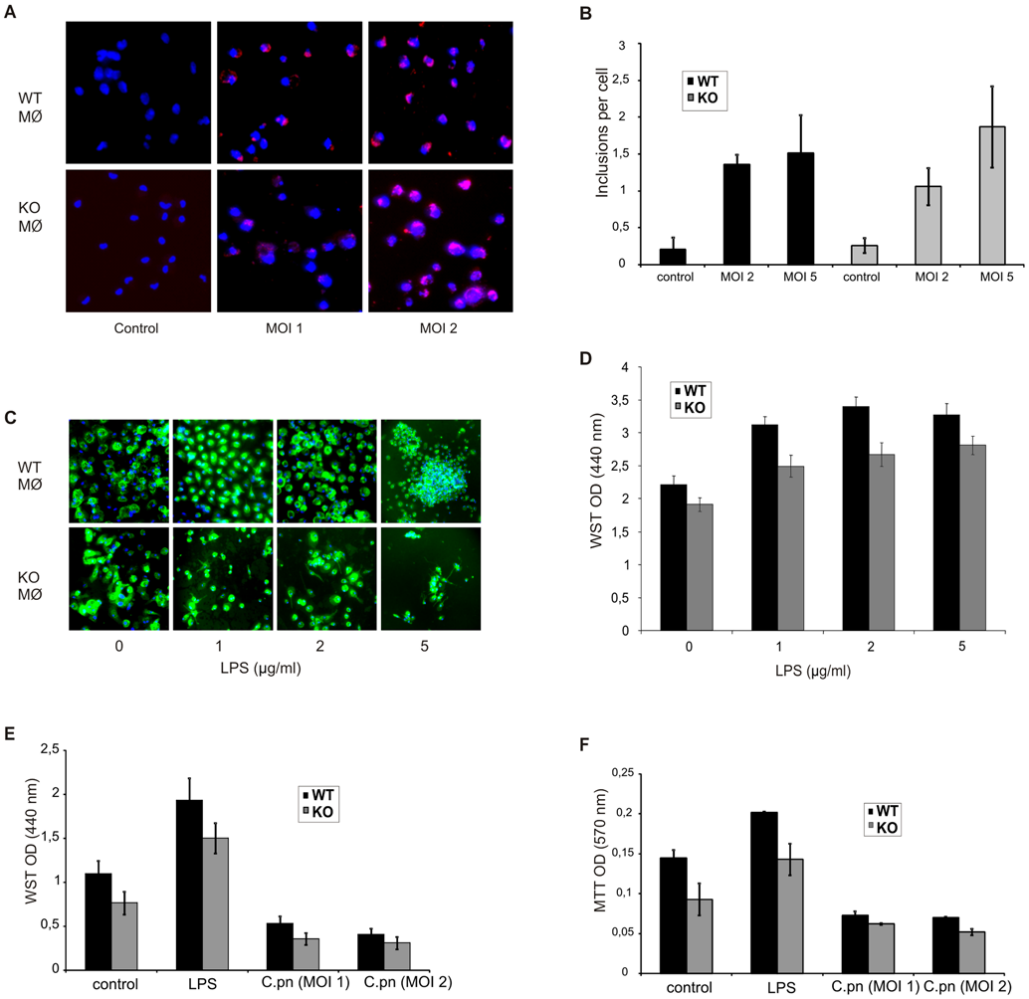
Impaired growth of cIAP-1 KO macrophages. A. Peritoneal macrophages from wildtype and ciap-1 KO mice were harvested and cultured in antibiotic free RPMI-1640 medium over night. Next day the macrophages were infected with *C. pneumoniae* at different MOI. 72 h post infection, the macrophages were fixed with chilled absolute methanol and stained with anti *C. pneumoniae* antibody. Shown here is the representative micrographs of the macrophage infected. B. Quantification of inclusions in macrophages. 1×10^4^ wildtype and cIAP-1 KO macrophages were cultured in 96 well plates in antibiotic free medium and infected with different doses of *C. pneumoniae*. The cells were fixed and stained with anti-*C. pneumoniae* antiserum. The inclusion in the macrophages were determined by automated microscopy as described in Materials and Methods. Data are represented as mean±SEM from three independent experiments. C. Quantification of Mac-1+(CD11b+) peritoneal macrophages. The macrophage from wildtype and cIAP-1 macrophage were treated with increasing doses of LPS and Mac-1+peritoneal macrophage were compared among wildtype and KO mice. Shown here is the immune fluorescence picture of Mac-1+macrophages (green) and DNA (blue) and analyzed by immunofluorescence microscopy. D. Mouse peritoneal macrophages from wildtype and cIAP-1 KO mice were stimulated *ex vivo* with different doses of LPS and their survival was measured 48 h later by WST assay. E. Toxicity of *C. pneumoniae* infection on macrophages. Metabolic activity of infected was determined by standard WST assays as described in Supporting Materials and Methods S1. Data are represented as mean of OD±SEM from three independent experiments. F. The survival of macrophages under infection experiments was measured by standard MTT assays as described in Supporting Materials and Methods S1. Data are represented as the mean OD±SEM from three independent repeats.

**Figure 6:**
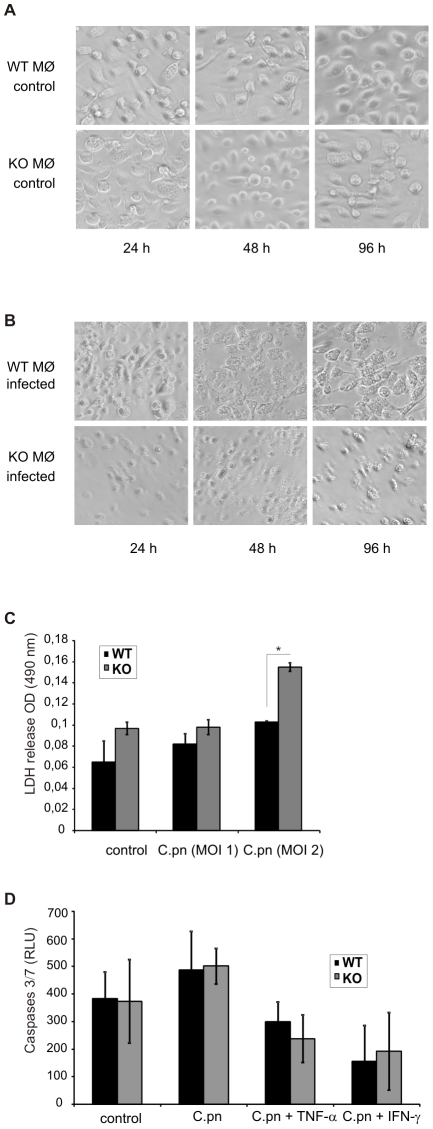
Cytotoxicity of *C. pneumoniae* infection on macrophages. A and B. Phase contrast pictures of non-infected (control) and infected macrophages from wildtype (WT) and cIAP-1 KO (KO) mice were taken 24 h, 48 h and 96 h post infection at 40×magnification. Shown are representative pictures from one out of three independent experiments. C. The culture supernatants from macrophages infected as in figure 5 or 6A,B were taken and cytotoxicity of infection was compared quantitatively by LDH release at 72 h post infection using the LDH cytotoxicity detection kit (Roche). D. Both wildtype and cIAP-1 KO macrophages were infected and stimulated with TNF-α and IFN and activity of caspase-3 and -7 was quantified using Caspase-Glo^®^ luminescent assay at 24 h post infection. * p≤0.05.

The depletion of macrophages from the lungs of infected cIAP-1 KO mice raised the question whether infection directly affects survival of macrophages. We therefore investigated the role of cIAP-1 in the survival and proliferation of macrophages in response to *C. pneumoniae* infection and/or mitogenic stimuli. To test whether cIAP-1 affects the infection efficiency, macrophages from wildtype and cIAP-1 KO animals were infected and the inclusions were detected by immunofluorescence microscopy. Inclusions could be detected at similar quantities in wildtype and KO macrophages ruling out a major role of cIAP-1 in infection of *C. pneumoniae* (Fig. 5A,B). We next tested whether infection or LPS treatment affected the proliferation of Mac-1-positive macrophages differently. Macrophages were treated with LPS and the metabolic activity was measured using the WST assay. If treated with LPS, cIAP-1 KO macrophages exhibited less metabolic activity compared to wildtype macrophages (Fig. 5C,D). A decrease in metabolic activity was observed upon infection of both, wildtype and KO macrophages with *C. pneumoniae* (Fig. 5E). A similar result was obtained when the survival of treated and infected macrophages was measured using the MTT assay (Fig. 5F). We also observed the loss of infected cIAP-1 KO macrophages from the cell culture plate after prolonged infection (Fig. 6A,B). To further characterize the reason for the cell loss, LDH release and caspase 3/7 activation was measured to assay necrotic and apoptotic cell death, respectively. Whereas caspase 3/7 was not increased in KO macrophages compared to wildtype cells, a small but significant (p≤0.05) increase in LDH activity could be measured in the culture supernatants (Fig. 6C,D), suggesting a necrotic type of cell death induced by *C. pneumoniae* infection.

### Loss of functions in immune cells from cIAP-1 KO

**Figure 7:**
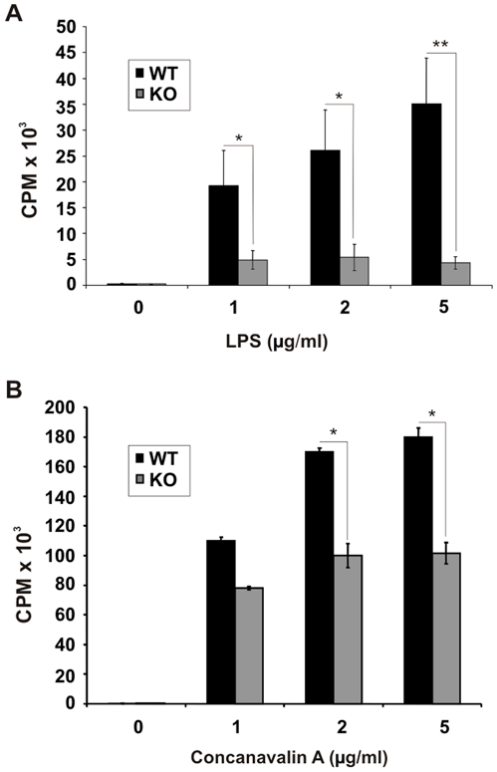
Impaired stimulatory potential in splenocytes from cIAP-1 KO mice. A and B. 1×10^6^ PMC (A) and splenic T cells (B) from wildtype and cIAP-1 KO mice were stimulated with different doses of LPS and Con A, respectively, and the stimulatory potential was measured by ^3^H-thymidine incorporation. Data represent the mean of counts per minutes CPM±SEM from three independent experiments. * p≤0.05; ** p≤0.01.

To find out whether the defect observed was specific for macrophages we tested other immune cells for their response to mitogenic stimuli by ^3^H-thymidine uptake assays. Treatment of splenic polymorphonuclear cells (PMC) and T cells (for details see Supporting Materials and Methods S1) with LPS and Concanavalin A, respectively, resulted in an enhanced proliferation of both wildtype and cIAP-1 KO cells. However, cIAP-1 KO leukocyte were strongly compromised in their proliferation compared to wildtype cells (Fig. 7A,B).

In conclusion, these data demonstrate the involvement of cIAP-1 in the control of *C. pneumoniae* infection and the integrity of the innate immune functions of mouse macrophages and splenocytes.

## Discussion

*C. pneumoniae* infection induces the expression and increased stability of *inhibitor of apoptosis proteins* IAPs, which confer resistance of infected cells to apoptosis induced by different stimuli [14]. Based on this observation and since induction of apoptosis has been suggested a potential strategy to activate anti-bacterial responses required for the resolution of bacterial infections [28], we assumed that inhibiting apoptosis of infected cells would ensure bacterial survival. Interfering with infection-induced anti-apoptotic signaling should confer resistance against bacterial infection. In contrary to our expectations, cIAP-1 KO mice were clearly sensitized for pulmonary infection with *C. pneumoniae*, strongly supporting a role of cIAP-1 in the immune response against these obligate intracellular bacteria.

Accumulating evidence suggests that cIAP-1 and cIAP-2 are involved in signal transduction including the NF-κB pathway [29]–[32]. Recent data demonstrated a key role of these IAPs in the control of TNF-α-induced NF-κB signaling. However, cIAP-1 and cIAP-2 exert redundant functions in this signaling pathway [29]. Moreover, the cIAP-1 knockouts were initially reported to have no overt phenotype, because of compensation by the up-regulation of cIAP-2 in various tissues and cells such as spleen, thymus and MEFs [33]. We observed the up-regulation of cIAP-2 in lung tissue, but not in macrophages of cIAP-1 knockout mice. Due to the clear phenotype of the cIAP-1 knockout mice, we postulate a non-redundant role of cIAP-1 in chlamydial lung infection. This does not exclude a function of cIAP-2 in chlamydial lung infection since both may be organized in functional complexes [15].

*C. pneumoniae* infection induces secretion of inflammatory mediators like IFN-γ in host macrophages, which is crucial for their anti-bacterial defense via induction of TNF-α [34]. It was therefore expect that increased titers of IFN-γ in the lungs of infected cIAP-1 KO mice results in the increased production of TNF-α. However, infected cIAP-1 KO mice had lower TNF-α titers than wildtype mice. This could be due to either the depletion of alveolar macrophages or their reduced recruitment to the lungs of infected cIAP-1 KO mice. These assumptions are supported by our comparative histo-pathological analyses of lungs from infected KO and wildtype animals (Fig. 2A). We found an increased sensitivity of cIAP-1 KO macrophages for high concentrations of TNF-α *in vitro* (not shown) compared to macrophages from wildtype animals. It is thus possible that macrophages from cIAP-1 KO mice undergo apoptotic cell death in the inflamed lung tissue, as suggested for cIAP-2 knockout mice [24]. However, enhanced macrophage apoptosis alone probably does not account for the increased infection of the cIAP-1 KO mice we observed, because macrophage apoptosis in the fully immune competent animal rather supports the clearance of lung infections [28]. Furthermore, we could not observe the sensitization for apoptosis of macrophages from KO versus wildtype animals upon infection with *C. pneumoniae* or treatment with different stimuli (Fig. 6B and S3). We therefore assumed a defect in the innate immune response was responsible for the reduced macrophage numbers and increased bacterial load of infected animals.

Among various virulent factors, *C. pneumoniae* LPS and HSP60 are well known stimulators of macrophage-mediated inflammatory responses which manifest a strong anti-bacterial defense [35], [36]. In line with a dysfunction of the cIAP-1 KO macrophages in these signaling pathways, LPS treatment or *C. pneumoniae* infection induced the production of TNF-α in the supernatant of wildtype macrophages whereas the response of knockout macrophages was reduced. A similar defect has been observed in cIAP-2 KO mice, which showed a profound resistance against LPS-induced inflammatory responses [24]. However, cIAP-1 KO mice were not only affected in LPS-triggered signaling pathways leading to TNF-α secretion. The response to IFN-γ treatment was also strongly reduced compared to wildtype macrophages, demonstrating an impaired IFN-γ induced signaling to TNF-α production in the cIAP-1 KO macrophages. Since TNF-α is one of the central cytokines involved in anti-chlamydial defense in both epithelial cells and macrophages [37], [38], the reduced production of TNF-α in the lungs and macrophages of cIAP-1 KO mice is very likely one of the main reasons for the opportunistic survival of *C. pneumoniae* in the lungs of infected cIAP-1 KO mice.

Production of inducible NO is another potent mechanism by which macrophages kill bacterial pathogens in general [27], [39] and *C. pneumoniae* in particular [40], [41]. NO is generated by nitric oxide synthases (NOS). Of the three NOS isotypes found in mammalian cells, NOS2 also called iNOS (inducible or independent of elevated Ca^2+^) is most important in the context of infection, inflammation and immune regulation [27], [39]. We found a strong decrease in NO production in macrophages from cIAP-1 KO mice stimulated with LPS and TNF-α compared to wildtype cells. Interestingly, this effect was not observed if macrophages were treated with IFN-γ suggesting that cIAP-1 is involved in specific signaling pathways leading to the generation of NO. The block in NO production in cIAP-1 KO macrophages is probably upstream of iNOS induction since inducers which triggered a cIAP-1-dependent increase in NO (LPS, TNF-α) also induced iNOS expression. However, stimulation of NO in wildtype and cIAP-1 KO macrophages by IFN-γ remained comparable and redundant (Fig. 4), indicative of cIAP-1-independent stimulation of NO by IFN-γ. This is especially interesting since induction of murine iNOS by LPS also involves the IFN-γ pathway, as demonstrated previously in IFN-γ [42], IFN-γ receptor [43] and STAT-1 [44] deficient mice. Since cIAP-1 KO macrophages express normal amounts of the LPS receptors, CD14 and TLR4 (HP and TR, unpublished), we speculate that cIAP-1 acts downstream of LPS receptors and upstream of IFN-γ induction to up-regulate iNOS.

We found an increased resistance of cIAP-1 mice for endotoxic shock induced by LPS treatment. Mortality of KO and wildtype mice infected with *Salmonella* was, however, similar, suggesting unknown mechanisms besides endotoxin exposure causing toxicity in case of infection. *Salmonella* infection of macrophages is known to activate different pathways leading to cell death [45]. One of these, called pyroptosis because of its proinflammatory effect, is a caspase-1-dependent form of cell death dependent on the *Salmonella* type-three secretion system (T3SS) and effectors [46]. As caspase-1 KO mice are resistant to *Salmonella* infection [47], pyroptosis induced by *Salmonella* may not be influenced by cIAP-1 *in vivo*.

There are at least two different possible mechanisms by which cIAP-1 KO mice resist endotoxin shock. Firstly, increased sensitivity of LPS treated cIAP-1 KO macrophages to cell death (see Fig. 5) may lead to the depletion of macrophages upon induction of a systemic inflammatory response. As a consequence, a major source for proinflammatory cytokines is ablated leading to an attenuated immune response and protection from shock. This scenario has been suggested as mechanism for endotoxin shock resistance of cIAP-2 KO mice [24]. However, the finding of severe defects in TNF-α production by cIAP-1 KO mice and the induction of iNOS upon TNF-α and LPS challenge shifts the focus to NO as an effector of shock. Systemic TNF treatment provokes a lethal shock syndrome, in which cardiovascular collapse is centrally orchestrated by NO [48]. Several lines of evidence support an important role for the vasodilator NO in hypotension, a hallmark of septic shock [49], [50]. The reduced response of cIAP-1 KO macrophages and splenocytes to proliferate in response to LPS and Con A treatment may well contribute to the resistance of these animals for LPS-induced endotoxin shock.

These findings suggest that, besides their role in apoptosis regulation, IAPs play a prominent role in innate and adaptive cellular immunity.

## Materials and Methods

### Ethics statement

Animal testing was performed according to German Animal Protection law (TierSchG). The application for the experiments was reviewed by the responsible local authorities, Landesamt für Gesundheit und Soziales, Berlin, and approved (permission No. G0325/05).

### *C. pneumoniae* stock

*C. pneumoniae* strain VR1310 was propagated in HEp-2 cells. These cells were infected with *C. pneumoniae* and lysed by mechanical disruption with a rubber policeman 72 hour post infection. Bacteria were subsequently harvested by centrifugation at 500×*g* at 4°C for 10 min. The pellet was ruptured using glass beads and the lysates were centrifuged as before. The supernatants were removed and centrifuged at 45,000×*g* for 45 min at 4°C in a SS34 rotor (Sorvall Instruments) to pellet C*hlamydia*. The bacteria were resuspended in SPG (sucrose-phosphate glutamate) buffer and stored at −80°C.

### Non-invasive intratracheal infection of mice

A novel non-invasive spray method was established for infecting mice with *C. pneumoniae*. Mice were anesthetized by intraperitoneal injection of Ketamine (120 mg/kg) and Xylazine (16 mg/kg) and placed backwards on a small box. A microsprayer (Penn-Century, Philadelphia, PA) was placed oropharyngeally into the trachea with the help of a laryngoscope and a binocular light microscope, and mice were infected by aerosolizing 5×10^6^ bacteria in 25 µl SPG buffer into the airways. Control mice were kept on placebo and received the same amount of SPG buffer. Mice were routinely examined and the infectivity was monitored over the schedule of 21 days. The body weight of each mouse and other toxicological measurements were recorded over the course of infection.

### Lung infectivity assay

Bacterial load in the lung of infected mice was monitored by conventional lung infection assay [51]. The lungs of mice were kept separately in the infecting medium (RPMI+5% FCS), minced mechanically using a mesh and homogenized using a tissue homogenizer to obtain a suspension. The cellular debris and fibrous tissues were removed by passing the homogenate through a 40 µm cell strainer. The filtrate was centrifuged at 500×g for 10 min at 4°C and the resulting supernatant contained chlamydial elementary bodies. Fresh HEp2 cell monolayer was used to infect in 96 well plates by centrifugation at 700×g at RT for 1 h. After centrifugation plates were incubated in 5% CO_2_ at 35°C for 1 h before the medium was replaced with fresh RPMI containing 5% FCS, 1% Gentamycin and 1 µg Cycloheximide and the culture was kept in a CO_2_ incubator at 35°C further for 72 h.

### Immunostaining and automated microscopy

For staining the chlamydial inclusion, the cell monolayer was blocked with PBS+0.2% BSA for 1 h and incubated with mouse anti *C. pneumoniae* antibody (Ab-Cam) for 1 h at RT. After washing, cells were further incubated with Cy3-conjugated affinity purified goat anti mouse IgG (monoclonal) antibody for 1 h at RT. The cell nuclei were stained with Hoechst dye. Images were acquired by a Scan R automated microscope system (Olympus) using UPLSAPO lenses (10×magnification) as previously described [52]. Infectivity was calculated as the number of chlamydial inclusions per cell using the Scan R-Analysis software.

### Histopathology of lung tissue

For histopathological analysis, mouse lungs were cut in 5 mm long pieces and fixed with 4% PFA for 1 h at RT. The tissues were washed, air dried and blocked by placing tissues in cryo-stat medium at −20°C. Ten µm sections were cut in a cryotome (Leica) and air dried in a closed box at 4°C over night. The next day, these sections were blocked with 2% FCS/TBS for 1 h at RT. The sections were probed with rabbit anti- *Chlamydia* LPS and anti mouse CD68 markers to stain *C. pneumoniae* and macrophages, respectively. After 3 washes with TBS at RT, the sections were incubated with anti rabbit Cy3-conjugated IgG for *C. pneumoniae* and anti rat Alexa 488 for macrophages for 1 h at RT. Lung epithelial cells were visualized by staining them with Hoechst dye for 5 min at RT. The sections were washed three times with TBS, rinsed shortly with distilled water and mounted on coverslips. The sections were analyzed using fluorescence microscopy under 40×magnification.

### Total nitrite production

Supernatants from macrophages were used to determine NO as nitrite (a stable form of NO) by the standard Griess reagent assay. Equal volumes of the culture supernatants and Griess reagent (1% sulphanilamide/0.1% N-(naphthyl) ethylene-diaminedihydrochloride in 1∶1 ratio) were mixed and absorbance was measured at 550 nm by Spectra max spectrometer (Molecular Devices). The amount of nitrite produced in samples was calculated against a NaNO_2_ standard curve by using the SPF program.

### ELISA for cytokines

TNF-α and IFN-γ titers in the lung homogenates and/or macrophages culture supernatants were measured by using an ELISA kit (BD Pharmingen, USA). Polystyrene plates (Maxisorp, Nunc, USA) were coated with monoclonal anti-cytokine antibody and incubated overnight at 4°C. The plates were washed and blocked with PBS+10% FBS for 1 h. Recombinant cytokine standards and samples were incubated for 2 h at RT. After washing, plates were further incubated with biotinylated detection antibody for 1 h at 25°C. Substrate solution was added and plates were incubated for 10–30 min at RT. The reaction was stopped and the plates were read at 492 nm by a multiwell spectrometer, Spectra max 250 (Molecular Devices). Cytokine concentrations in samples were calculated by SPF software using a recombinant cytokine standard curve.

### Westernblotting for IAPs

Lungs of KO and WT animals were washed with PBS and homogenized in 3 volumes RIPA buffer (10 mM Tris/HCl [pH 7,5], 130 mM NaCl, 1% Triton X-100, 0,02% SDS, phosphatase inhibitor [PhoSTOP], protease inhibitor [complete]). After incubation on ice for 1 h, the lysates were sonificated for 10 sec and cleared by centrifugation for 30 min at 4°C and 14,000 rpm. The middle layer containing proteins was centrifuged as above for additional clearing and protein amounts were determined by Bradford assay. For detection of XIAP, lysates were incubated with Sepharose G beads over night at 4°C to reduce serum IgGs. 2×Laemmli buffer (100 mM Tris/HCl [pH 6,8], 20% glycerin, 4% SDS, 1,5% 2-mercaptoethanol, 0,2% bromphenol blue) was added to the lysates and boiled at 95°C for 5 min. Protein lysates were separated by SDS-PAGE and transferred to a PVDF membrane (GE Healthcare). After blocking for 1 h with 5% skimmed dry milk in TBS containing 0.5% Tween-20 (TBST), the membrane was decorated over night at 4°C with the following primary antibodies: rabbit polyclonal cIAP-2 (H-85) and beta-Tubulin (H-235) (Santa Cruz) and mouse monoclonal XIAP (BD Transduction). Antibody-antigen complexes were detected by HRP-linked donkey anti-rabbit or sheep anti-mouse secondary antibodies (GE Healthcare).

### Western blotting for iNOS

Peritoneal macrophage treated with various inflammatory inducers (LPS, IFN-γ and TNF-α) were lysed in 50 mM Tris-HCl (pH 7.4), 150 mM NaCl, 2 mM EDTA, 1% Nonidet P-40, and protease inhibitor mixture and sonicated. The lysate was centrifuged at 14,000 rpm for 20 min at 4°C to separate cytosol and particulate fraction. Protein concentration was determined by the Bradford method (Bio-Rad, Munich, Germany). Proteins (10 µg for each lane) were separated on 7.5% SDS-PAGE and blotted on PVDF membrane by wet electroblotting. Blots were blocked overnight at 4°C with 5% non-fat dry milk in TBS-T at pH 7.5 (20 mM Tris base, 137 mM NaCl, and 0.1% Tween 20) and then incubated for 2.5 h with anti-iNOS (BD Pharmingen, Heidelberg, Germany) followed by the HRP conjugated secondary antibody. Blots were developed by ECL (Amersham, Life Sciences, Freiburg, Germany) and actin was used as a loading control for normalization.

### Splenocyte primary cell culture and proliferation

The spleens from both wildtype and cIAP-1 KO mice were excised aseptically and a single cell suspension was prepared as previously described [53]. The spleens was kept in serum free RPMI 1640 medium and crushed between two frosted-end slides. The splenic homogenate was filtered through 40 µM cell strainer for the removal of tissue and debris. The suspension was carefully transferred to a 15 ml conical centrifuge tube and centrifuged at 1,500 rpm for 10 min, 4°C. RBCs were removed by RBC lysing buffer (0.1 M NH_4_Cl). Lymphocytes were separated from adherent PMN and monocytes by adherence method. For that purpose the single cell suspension so prepared was incubated at 37°C for 3 h and floating lymphocytes were collected, centrifuged at 1,500 rpm, 10 min at 4°C and resuspended in RPMI 1640 medium supplemented with 10% heat-inactivated FBS, 15 mM HEPES buffer, 2 mM L-glutamine and 1% Gentamycin. The viable lymphocytes were counted by trypan blue dye exclusion method. These lymphocytes were stimulated with Con A for inducing their proliferation. The PNM cells were separated from lymphocytes in similar way and stimulated with LPS for inducing their proliferation in flat bottom 96-well polystyrene microtiter and their proliferation was measured by tritiated thymidine (^3^H-Td) uptake method. Cells after 72 h post treatment were pulsed with 0.5 µCi ^3^H-Td (18.5kBq) for 18 h and harvested on the glass fiber filter mats by using cell harvester. Radioactivity was counted by using liquid scintillation counter (Perkin Elmer).

### Experimental endotoxin shock

For inducing endotoxin shock, both wildtype and cIAP-1 KO mice (8–10 wk old, 5 per group) were administered various (sub- and supra-lethal) doses of bacterial endotoxin (LPS; *Salmonella* Typhimurium Sigma) in 0.2 ml volume of non-pyrogenic saline, intraperitoneally. Control mice were administrated the same volume of normal non-pyrogenic-saline. Survival of wildtype and cIAP-1 KO mice was compared during the observation period of one week.

## Supporting Information

Materials and Methods S1 (0.06 MB DOC)

Figure S1 Infection of mouse lungs with Chlamydophila pneumoniae. The histopathological analysis of lungs was performed to validate the novel non-invasive intra-tracheal infection method. Animals were infected and lungs were excised aseptically at different times post infection. 5 µm cryo-sections of lungs tissue of infected and control mice were fixed and stained for the presence of C. pneumoniae antigen (red) and Mac-1+macrophages (green). The lung epithelial cells were counter stained with DAPI (blue). The sections were analyzed by epi-florescent microscopy. Pictures shown here are typical examples of sections analyzed from three independent repeats.
(1.30 MB TIF)

Figure S2 Disruption of IAP levels in cIAP-1 KO macrophages. (A) Mac-1+macrophage from both WT and cIAP-1 KO mice were isolated. Total protein lysates were prepared from 1×10E6 macrophages as described and 20 µg of protein from WT and cIAP-1 KO macrophage were separated by SDS-PAGE and the blotted onto PVDF membrane. Levels of IAPs and beta-actin (loading control) in these macrophages were detected by immunoblot analysis using cIAP-1 (BD transduction), rabbit polyclonal antiserum cIAP-2 (H-85) (Santa Cruz) and mouse monoclonal XIAP (BD Transduction). (B) PCR genotyping of lung tissue from wildtype and cIAP-1 KO mice. DNA extracted from lung tissue of wildtype (WT) or cIAP-1 KO mice (KO) was amplified with primers that bind to a sequence within exon 1 of the cIAP-1 gene (ex1) or to a sequence in the neomycin gene (neo) that was inserted into exon 1 of the cIAP-1 gene to generate KO mice (neo).
(0.42 MB TIF)

Figure S3 No increased apoptosis of ciap-1 KO macrophages upon treatment with stimuli. A and B. 1×106 macrophages from both wildtype and cIAP-1 KO mice were isolated and cultured as described. Apoptosis was evaluated by determining caspases-3/7 activity by the Caspase-Glo® luminescent Assay. Macrophages were stimulated with various inflammatory mediators (TNF-alpha: 50 ng/ml; IFN-gamma: 50 ng/ml; LPS: 5 µg/ml; Sodium nitroprusside (SNP): 300 nM) and their response was monitored after 24 h (A) and 48 h (B) post treatment. Neither of the treatments elicited an apoptotic response in wildtype or cIAP-1 KO macrophages. Data are represented as mean of luminescence±SEM from two independent experiments with similar results. (C) Determination of macrophages viability. Macrophages treated with inflammatory cytokines and their survival was measured by MTT dye reduction method. The macrophages under above treatments were incubated with MTT dye and The amount of purple formazon crystals formed as a result of its reduction was measured at 570 nm by a spectrometer, Spectra max 250 (Molecular Devices, Munich, Germany). Data are represented as mean OD±SEM from three independent experiments.
(0.25 MB TIF)

Figure S4 No effect of cIAP-1 in the control of Salmonella infection and toxicity Intravenous infection of C57BL/6 and KO cIAP-1 mice with Salmonella Typhimurium. C57BL/6 and KO cIAP-1 mice were infected with 500 colony forming units (cfu) Salmonella Typhimurium SL1344 via a tail vein. A. cfu per spleen were determined 5 days post infection. Bacterial load is equivalent in both C57BL/6 and KO cIAP mice. B. Survival was monitored over one week after infection. All mice were dead 6 days post infection without any significant difference in C57BL/6 or KO cIAP-1 mice.
(0.16 MB TIF)

Table S1 (0.03 MB DOC)

Table S2 (0.03 MB DOC)
