# Supplementary material for: cIAP-1 Controls Innate Immunity to C. pneumoniae Pulmonary Infection
Source: PLoS One. 2009 Aug 6;4(8):e6519. doi: 10.1371/journal.pone.0006519 (PMC2716518; doi:10.1371/journal.pone.0006519)
Supplement: Materials and Methods S1 — (0.06 MB DOC) [file pone.0006519.s001.doc]

**Supporting information**

**Supporting Materials and Methods**

**Reagents and antibodies**

LPS (*Salmonella tympanum)*, Gentamycin, MTT and NaNO_2_ were purchased from Sigma (Taufkirchen, Germany). TNFα OptEIA^TM^ ELISA kit was purchased from BD Pharmingen (Heidelberg, Germany). (^3^H)-Thymidine was purchased from Amersham (Freiburg, Germany). DNA easy kit and SYBR green PCR reaction mixture were purchased from Qiagen GmbH (Hilden, Germany). *C. pneumoniae ompA* gene primers were purchased from Invitrogen (Karlsruhe, Germany). Red Taq Polymerase was purchased from Sigma. Anti *C. pneumoniae* LPS antibody and mouse anti *C. pneumoniae* antibody (IgG) were purchased from Abcam (Cambridge, UK). Anti mouse CD68 antibody was purchased from BD Pharmingen (Heidelberg, Germany). Cy3-conjugated affinity purified goat anti mouse IgG and anti rabbit Cy3 antibody were purchased from Jackson laboratories (through Dianova, Hamburg, Germany). Alexa fluor 488 conjugated antibodies were purchased from Invitrogen, (Karlsruhe, Germany). Ketavet was purchased from Pharmacia GmbH (Karlsruhe, Germany) and Rompum were purchased from Bayer vital GmbH (Leverkusen, Germany).

**cIAP-1 knockout mice**

cIAP-1 knockout mice (B6.129P2 - cIAP1 tm1) in the lab were generated at the ‘The Campbell Family Institute for Breast Cancer Research, Toronto, Canada’ as described earlier [1]. These mice were established, housed and bred at the MPIIB animal housing facility, Berlin in a pathogen-free environment. All experiments were performed in accordance with the guidelines of the institutional animal ethical committee. For infection experiments, 8 - 11 weeks old cIAP-1 and conisogenic wildtype C57BL/6 mice (n = 5 per group) were kept separately under aseptic conditions and provided with 12 h photoperiod and fed with pathogen free food and water *ad libidum.*

**Genotyping of WT and cIAP-1 KO lung tissue**

DNA extraction from lung tissue of cIAP-1 KO and WT mice was performed with a DNeasy Blood & Tissue kit (QIAGEN) according to the manufacturer`s instructions. 80 ng of extracted DNA was utilized for PCR amplification with the following primers: forward primer recognizing exon 1 of cIAP-1 gene of WT (ex1): GGTCACATGAACTCTTCTGGGTAACCTG, forward primer recognizing the neomycin gene of KO (neo): GCTCATTCCTCCCACTCATGATCTATAG, reverse primer for WT and KO: GGTGAGGAACTGCTGTAGTCAAGATTTG. PCR was performed in a thermocycler as follows: 2 min at 94°C, 35 cycles with 94°C for 45 sec, 60°C for 60 sec, and 72°C for 90 sec, and a final step at 72°C for 5 min. PCR products were run on a 1% agarose gel and visualized with GelRed nucleic acid stain (Biotrend).

**Polymerase Chain Reaction**

*C. pneumoniae* in the animal tissue was monitored by amplification of the *ompA* gene by nested and quantitative real time PCR (qRT-PCR) as described [2]. One μg of DNA from each lung sample was used to detect *C. pneumoniae* by nested PCR. The primers used for the amplification of *ompA* gene had the following sequences: HL-1: 5’-GTTGTTCATGAAGGCCTACT-3’, HM-1: 5’-GTGTCATTCGCCAAGGTTAA-3’; HR-1: 5’-TGCATAACCTACGGTGTGTT-3’ (Invitrogen, Karlsruhe, Germany). PCR was carried out in 25 μl volume containing 50 mM of deoxynucleotide triphosphate (dNTPs), 1 U Red Taq polymerase, 5 mM Mg^2+^, 1 mM Tris-HCl, and 50 mM KCl, pH 8.3 and 0.5 mM of HL-1 and HR-1 primer pairs. The PCR reaction consisted of an initial denaturation at 94^o^C for 15 min followed by 40 repeats of denaturation at 94°C for 1 min, annealing at 55^o^C for 1 min, and primer extension at 72^o^C for 7 min. One μl of the product from this reaction was nested for second reaction and amplified by using HR-1 and HM-1 primers. These two reactions yielded 437 and 229 bp products corresponding to the VD2 and VD4 fragment of *omp*A gene respectively when analyzed on agarose gel. The *gapdh* gene was used as positive control to validate the reaction. Presence of these two products was rate as successful infection.

To quantify the amount of *omp*A gene present in lungs of infected mice, 10 ng of the genomic DNA from each lung sample was amplified by SYBR green dye method under similar conditions. The fluorescence intensity of each sample amplified was measured and the amount of *omp*A gene in each lung sample was quantified by using SDS software, Applied Biosciences version 2.2.2 (Applied Biosciences, Darmstadt, Germany). All the values obtained for *ompA* gene were normalized against mouse GAPDH internal control.

**Peritoneal macrophage isolation**

Mouse peritoneal macrophages were established by the procedure described elsewhere [3]. Briefly, 4% Brewer’s thioglycolate medium (Becton Dickinson, Heidelberg, Germany) was injected into the peritoneal cavity of each mouse. Peritoneal exudate was obtained 72 h after injection by flushing the peritoneal cavity with ice-cold serum free RPMI 1640 medium using a 22G needle. Peritoneal lavage was pooled and centrifuged in a 50-mL conical centrifuge tube at 1500 rpm for 10 min at 4^o^C and the pellet was resuspended in RPMI 1640 medium supplemented with 10% heat-inactivated fetal calf serum, 15 mM HEPES buffer, 2 mM L-glutamine and 1% Gentamycin. Cells were counted and checked for viability by the trypan blue dye exclusion method. 1 × 10^6^ cells per well were cultured in a final volume of 200 µl in flat-bottom 96-well polystyrene microtiter plates (Costar, Sdriphol-Rijk, Netherlands) and incubated at 37^o^C for 3 h for adherence. Non-adherent cells were removed by washing with serum free RPMI 1640 medium. Cells were identified by flow cytometry using antibody against Mac-1 (BD Pharmingen, Heidelberg, Germany).

**Infection of peritoneal macrophages**

Peritoneal macrophages isolated from mice were infected with *C. pneumoniae* *ex vivo* in RPMI containing 5% FCS. Cells were centrifuged at 700 × g for 1 h at RT for infection. After centrifugation, the cells were incubated for 1 h in a CO_2_ incubator at 37^o^C. After 1 h of incubation the medium was replaced with RPMI containing 5% FCS, 1% Gentamycin and 1 µg/µl of Cycloheximide. The cultures were incubated further for different time intervals at 37^o^C in CO_2_ incubator at 95% humidity.

**Caspases 3/7 activity**

Caspases-3 activity was measured by Caspase-Glo^TM^ 3/7 Assay kit® (Promega, Madison, USA). One million macrophages in each experimental group were cultured and treated with various inflammatory mediators, LPS, TNFα and IFN-γ, and cultured for different time periods. The cells were then collected and incubated with Caspase GLO reagent for 1-2 h in opaque 96 well plates. The amount of cleaved caspase-3 in each group was determined quantitatively by luminescence measurement.

**Cell survival and stimulation**

Survival of mouse macrophages and splenocytes from various experimental conditions was tested by MTT dye reduction method. Cells were incubated with yellow MTT [3-(4,5-dimethylthiazol-2-yl)-2,5-diphenyltetrazolium bromide (Sigma, Taufkirchen, Germany)]. The amount of purple formazan crystals formed was measured at 570 nm by a spectrometer, Spectra max 250 (Molecular Devices, Munich, Germany). Stimulation of mouse peritoneal macrophages and splenocytes by various treatments and *C. pneumoniae* infection was determined by WST-1 assays (Roche Diagnostics, Mannheim, Germany). Unless otherwise specified, 10 μl of reagent was added to a 0.2 ml volume of cell culture and incubated for 1 h and the absorbance was measured at 440 nm by a spectrometer, Spectra max 250 (Molecular Devices, Munich, Germany).

**Infection of mice with *Salmonella* Typhimurium and determination of cfu in the spleen**

*Salmonella* Typhimurium SL1344 was plated from frozen stock on LB agar (90 µg/ml streptomycin), grown over night at 37°C. From the plate 10 colonies were inoculated into 5.0 ml LB liquid medium (90 µg/ml streptomycin), grown over night (37°C, 200 rpm). Next morning 5.0 ml fresh LB liquid medium (90 µg/ml streptomycin) were inoculated 1:100 with over night culture and grown to an optical density OD_600_=1.0 (late log phase). 2.0 ml of the culture were spun down (table centrifuge; 14,000 rpm), supernatant carefully removed and washed three times by resuspending the pellet in endotoxin free PBS to remove endotoxin released by the *Salmonella* into the medium to avoid septic shock in mice. The suspension was adjusted to 500 cfu/100 µl and 100 µl of this suspension injected into the tail-vein of C57BL/6 and KO cIAP-1 mice. Survival was monitored over two weeks and cfu in the spleen determined five days post infection. To determine cfu in the spleen, mice were sacrificed, the spleen homogenised in 1.0 ml PBS containing 1.0% Triton X-100 using the punch of a 10 ml syringe and several dilutions plated on LB agar (90 µg/ml streptomycin) and the cfu calculated after over night incubation (37°C).

**References**

1. Conze DB, Albert L, Ferrick DA, Goeddel DV, Yeh WC et al. (2005) Posttranscriptional downregulation of c-IAP2 by the ubiquitin protein ligase c-IAP1 in vivo. Mol Cell Biol 25: 3348-3356.

2. Arno G, Kaski JC, Smith DA, Akiyu J, Zal B et al. (2005) Detection of Chlamydia pneumoniae in atherosclerotic tissue: a comparative study of PCR and immunocytochemistry. Br J Biomed Sci 62: 155-160.

3. Edelson PJ, Cohn ZA (1976) 5'-Nucleotidase activity of mouse peritoneal macrophages. I. Synthesis and degradation in resident and inflammatory populations. J Exp Med 144: 1581-1595.
