# Supplementary material for: cIAP-1 Controls Innate Immunity to C. pneumoniae Pulmonary Infection
Source: PLoS One. 2009 Aug 6;4(8):e6519. doi: 10.1371/journal.pone.0006519 (PMC2716518; doi:10.1371/journal.pone.0006519)
Supplement: Table S1 — (0.03 MB DOC) [file pone.0006519.s006.doc]

**Table S1: Detection of *C. pneumoniae* in organs of mice.**

|  | **3 days post infection** | | | **10 days post infection** | | | **20 days post infection** | | |
| --- | --- | --- | --- | --- | --- | --- | --- | --- | --- |
| **experimental group** | **Lung Liver Spleen** | | | **Lung Liver Spleen** | | | **Lung Liver Spleen** | | |
| **WT control (n=5)**  **WT infected (n=5)** | - / -  + / + | - / -  - / + | - / -  - / + | - / -  + / + | - / -  - / + | - / -  - / + | - / -  + / + | - / -  - / - | - / -  - / - |

*Chlamydia* was detected by nested PCR in different organs and at different time points post infection (for details see Supplemental Materials and Methods). -/-: both PCRs negative; -/+: 1. PCR negative/2. PCR positive; +/+: both PCRs positive.
