# Supplementary material for: cIAP-1 Controls Innate Immunity to C. pneumoniae Pulmonary Infection
Source: PLoS One. 2009 Aug 6;4(8):e6519. doi: 10.1371/journal.pone.0006519 (PMC2716518; doi:10.1371/journal.pone.0006519)
Supplement: Table S2 — (0.03 MB DOC) [file pone.0006519.s007.doc]

**Table S2: Detection of *C. pneumoniae* in lungs of mice.**

|  | **3 days post infection** | **10 days post infection** | **20 days post infection** |
| --- | --- | --- | --- |
| **WT control (n=5)**  **cIAP-1 KO control (n=5)** | - /-  - / - | - / -  - / - | - / -  - / - |
| **WT infected (n=5)**  **cIAP-1 KO infected (n=5)** | + / +  - / + | + / +  + / + | + / +  + / + |

The table shows the efficacy of lung infection in both wildtype and cIAP-1 KO mice. For details see Supplemental Materials and Methods. *Chlamydia* was detected by nested PCR in different organs and at different time points post infection. -/-: both PCRs negative; -/+: 1. PCR negative/2. PCR positive; +/+: both PCRs positive.
